# E3 ligase Skp2-mediated stabilization of survivin contributes to radioresistance

**DOI:** 10.1038/s41420-025-02463-3

**Published:** 2025-04-07

**Authors:** Shiming Tan, Ruirui Wang, Jinglin Fang, Ming Yi, Pengfei Guo, Shuangze Han, Xiaoying Li, Yu Gan, Jinzhuang Liao, Xinfang Yu, Wei Li

**Affiliations:** 1 Department of Radiology, The Third Xiangya Hospital of Central South University, Changsha, China; 2 Department of Haematology, The Third Xiangya Hospital of Central South University, Changsha, China; 3 School of Stomatology Hunan University of Chinese Medicine, Changsha, China; 4 Key Laboratory of Carcinogenesis and Cancer Invasion of Chinese Ministry of Education, XiangYa Hospital, Central South University, Changsha, China; 5 Key Laboratory of Carcinogenesis of National Health Commission, Cancer Research Institute and School of Basic Medical Science, Xiangya School of Medicine, Central South University, Changsha, China

**Keywords:** Oral cancer, Cancer therapeutic resistance, Radiotherapy

## Abstract

Oral squamous cell carcinoma (OSCC) is a frequently occurring neck and head malignancy. Therapies for OSCC are improving, but radiotherapy resistance remains a major clinical challenge. Here, we found that the S-phase kinase-associated protein 2 (Skp2) is overexpressed in OSCC cells and tissues. Knockdown of Skp2 significantly increased the radiotherapy sensitivity of OSCC cells. Further potential mechanisms suggest that Skp2-deficient restoration of radiotherapy sensitivity in OSCC cells may induce intrinsic apoptosis through inhibition of the Akt/Wee1/CDK1 axis, which inhibits Survivin phosphorylation and promotes its ubiquitination and degradation by FBXL7. Clinicopathologic histological analysis showed that Skp2 was positively correlated with the expression of p-Akt and Survivin in OSCC tissues. Furthermore, knockdown or inhibition of Skp2 overcame the radiotherapy resistance of OSCC cells. In conclusion, our study demonstrated that targeting the Skp2-Survivin axis could serve as an attractive and promising potential therapeutic target for radiotherapy sensitization in OSCC.

## FACTS

- Oral squamous cell carcinoma (OSCC) is a common malignancy in the head and neck region, radioresistance remains a significant clinical challenge in OSCC treatment.
- S-phase kinase-associated protein 2 (Skp2) is overexpressed in OSCC cells and tissues.
- Knockdown of Skp2 enhances the sensitivity of OSCC cells to radiotherapy by inducing intrinsic apoptosis through targeting the Akt/Wee1/CDK1 axis.
- Targeting the Skp2-Survivin axis may offer a promising therapeutic strategy for sensitizing OSCC to radiotherapy.

## Introduction

Oral squamous cell carcinoma (OSCC) develops in the epithelium of the oral mucosa, representing around 90% of oral malignancies [1–3]. In addition to genetic predisposition due to mutations, the development of squamous oral cancer is closely related to many bad habits, such as smoking and alcohol intake [4]. Radical surgery with adjuvant radiotherapy is the mainstay for patients with OSCC, but radiotherapy has still not achieved satisfactory results due to inter-individual differences in sensitivity to radiotherapy and acquired resistance [2, 5]. There is an urgent need to develop effective strategies for overcoming radioresistance in OSCC.

The ubiquitin-proteasome system (UPS) is a crucial pathway for intracellular protein degradation [6], regulating various life processes such as the cell cycle and signaling [7, 8]. S-phase kinase-associated protein 2 (Skp2), is a crucial F-box protein involved in UPS and contributes significantly to the pathogenesis of multiple diseases via its E3 ligase activity [9]. Skp2 has been shown to regulate multiple signalings, including cell cycle regulators such as P21 [10], P27 [11], P57 [12], cyclinE [13], Akt [14], YAP [15], and CRAM1 [16]. Furthermore, Skp2 is overexpressed in human cancers and correlates with poor prognosis [9, 14, 17, 18]. However, the mechanism regarding the role of Skp2 in OSCC radiotherapy resistance is not yet clear.

Survivin, first reported in 1997, is a 142 amino acid member of the inhibitor of apoptosis proteins (IAPs) family, with a molecular mass of approximately 16.3 kDa. It exhibits both proliferative and anti-apoptotic properties [19–21]. Research indicates that Survivin is primarily expressed in human embryonic tissues and various malignant tumors. Aberrant expression of Survivin has been observed in tumor cells, leading to anti-apoptotic effects [22]. Moreover, studies have shown a positive correlation between Survivin expression and the malignancy level in oral squamous cell carcinoma (OSCC) [23–25]. Therefore, targeting Survivin shows promise as a potential treatment for OSCC.

In the project, we observed that deleting Skp2 increased radiation-induced apoptosis and reduced Survivin protein levels. Our findings suggest that targeting the Skp2-Survivin axis could be an effective strategy to overcome radioresistance in OSCC.

## Method

### Cell lines and cell culture

The human oral squamous carcinoma cell lines, CAL27, SCC4 and SCC25, were obtained from the American Typical Culture Collection (ATCC, Manassas, VA). The medium used for the three cell lines was DMEM/F12 medium containing 10% fetal bovine serum (FBS). The culture environment was 37 °C, 5% CO_2_ and moderate humidity in a constant temperature incubator. The radioresistant OSCC cells, CAL27-IR and SCC25-IR were generated in our lab as described previously [26].

### Reagents & antibodies

The inhibitors, including MG132, cycloheximide (CHX), Necrostatin-1 (Nec-1), z-VAD-FMK and Chloroquine (CQ) from Selleck Chemicals (Houston, TX). Antibodies against Survivin (#2808; IB: 1:1000; IHC: 1: 2000), Skp2 (#2652, IB:1:2000, IHC: 1:1000), γ-H2AX (#9718; IB: 1:1000; IF: 1: 800), p-CDK1 Thr161 (#9114; 1:1000), p-Akt Ser473 (#4060; IB: 1:1000; IHC: 1: 50), α-Tubulin (#2125; 1:5000), VDAC1 (#4866; 1: 3000), Bax (#14796; 1:1000), Akt (#4691; 1:2000), anti-mouse IgG HRP (#7076; 1:10000), Flag-tag (#8146; 1:1000), cleaved-caspase 3 (#9664; IB: 1:1000; IHC: 1: 2000), p-Wee1 Ser642 (#4910; 1:1000), p-Survivin T34 (#8888; 1:1000), cytochrome C (#11940; 1:1000), cIAP2 (#3130; 1:1000), cIAP1 (#7065; 1:1000), Ub-k48 (#8081; 1:1000), β-actin (#3700; 1:1000) were obtained from Cell Signaling Technology, Inc. (Beverly, MA). Antibodies against Ki67 (#ab15580; IHC: 1:2000) and FBXL7 (#ab59149; 1:1000) were purchased from Abcam (Cambridge, UK).

### ShRNA/sgRNA cell line establishment

We constructed lentiviral vectors for gene knockdown/knockout using plasmid transfection. During the transfection process, HEK293T cells were used, and Lipofectamine 2000 (Invitrogen) and Opti-MEM serum-free medium were employed. After 6 h, the medium was replaced with fresh culture medium, and viral supernatant was collected 48 h later. For the construction of the shRNA/sgRNA cell line, target cells were transduced with the viral supernatant and 8 μg/ml poly-L-lysine (Sigma) was added to enhance transduction efficiency. 24 h after infection, 2 μg/ml puromycin (concentration should be determined through preliminary experiments) was added for selection, lasting for 5 days. Subsequently, cell proliferation was monitored, or short-term functional analysis was performed using the MTS assay. The following sgRNAs and siRNAs were used in the experiment: sgRNA#1 for Skp2: CCAGAGACCTTTAGCAGCTC; sgRNA#2 for Skp2: TCCCTCCAAAGGTGTTTCAT; siSurvivin (sc-29499), siFBXL7 (sc-62306), and siXIAP (sc-37508) were purchased from Santa Cruz Biotechnology.

### MTS assay

To analyze the effects of ionizing radiation (IR) on OSCC cells, cells were seeded in 96-well plates at a density of 3 × 10^3^ cells per well and cultured for 24 h. After incubation, cells were treated with or without IR. MTS reagent (#G3581, Promega, Madison, WI) was then added to each well, and the cells were incubated for 1–2 h. Cell viability was analyzed.

### Colony formation assays

As previously reported [27]. In the soft agar colony formation assay, the cell density was maintained at 8 × 10^3^ cells/well, and they were inoculated in 6-well plates containing Eagle’s basal medium. The colonies were observed and counted after 2 weeks of continuous incubation in a constant temperature incubator. The colony was counted with a light microscope.

### Caspase 3 activity assay

OSCC cells were collected and washed with PBS, followed by cell lysis using the lysis buffer provided in the Caspase 3 Assay Kit (#ab39383, Abcam). After centrifugation, the supernatant was collected, and the reaction buffer along with caspase 3 substrate (DEVD-AFC) was added. Fluorescent intensity was measured using a fluorescence microplate reader with excitation at 400 nm and emission at 505 nm to quantify caspase 3 activity.

### Western blotting (WB) assay

OSCC cells treated accordingly were given RIPA lysis buffer to obtain whole cell extracts (WCE). Protein concentrations were measured using the BCA Protein Assay Kit (#22328, Thermo Fisher Scientific), and then equal amounts of protein samples were separated on an SDS-PAGE gel and transferred to a PVDF membrane. After sealing with 5% skimmed milk for 60 min, the membrane was incubated with the primary antibody at 4 °C, followed by the secondary antibody (60 min) at room temperature. Target protein bands were detected with Enhanced Chemiluminescence Reagent (ECL) (#34579, Thermo Fisher Scientific).

### Co-immunoprecipitation (Co-IP) assays

Co-IP assays were performed according to established protocols [28]. Cells were treated with or without IR and cultured for 24 h. Cell lysates were prepared using IP lysis buffer (#87788, Thermo Scientific), and protein concentration was measured according to the manufacturer’s instructions. Protein A/G-agarose beads and the corresponding antibody were added and incubated at 4 °C for 24 h. The protein interaction was then determined by western blotting analysis.

### Ubiquitination analysis

The ubiquitination analysis, which were carried out according to the specific methodology in our published article [9]. The cell lysate obtained was sonicated, then heated in a metal bath to 95 °C and kept at that temperature for 15 min. Next, RIPA buffer containing 0.1% SDS was introduced and centrifuged at 16,000 x g for 10–15 min. The supernatant was carefully removed, combined with specific antibodies and agarose beads, and co-incubated overnight at 4 °C with spinning. The up-sampling buffer was then prepared the following day.

### Immunohistochemical (IHC) staining

IHC staining was performed following the previously reported [29]. Antigen retrieval from tissue sections was achieved using a sequence of xylene, ethanol, and 10 mM citrate. Following three washes with distilled water (ddH_2_O), a 3% H_2_O_2_ methanol solution was applied for 10 min to deactivate peroxidase. A blocking solution, consisting of 50% goat serum albumin in PBS, was prepared for blocking, followed by co-incubation with the primary antibody for 24 h at 4 °C. Subsequently, the sections were rinsed thrice with PBS and exposed to the secondary antibody for 45 min. Finally, positive staining was visualized using DAB, with Hematoxylin serving as the counterstain. The percentage of positive cells was scored as follows: 0, no positive cells; 1, ≤10% positive cells; 2, 10–50% positive cells; 3, >50% positive cells. Staining intensity was scored as follows: 0, no staining; 1, weak staining; 2, moderate staining; 3, dark staining. Comprehensive score = staining percentage × intensity. Skp2 expression levels were then determined based on this comprehensive score: a score of <1.5 indicated low expression, while a score of ≥1.5 indicated high expression.

### Clinical tissue sample collections

OSCC tumor tissues and the corresponding adjacent non-tumor tissues were from the Pathology Departments at Xiangya Hospital. Pathological samples of the patients included oral squamous carcinoma pathological tissue samples and paraneoplastic normal tissue samples (*n* = 74). The diagnosis and classification of patients were performed by the Department of Pathology of Xiangya Hospital according to the guidelines of the World Health Organization. All patients signed an informed consent form when they participated in this study.

### In vivo tumor growth

The in vivo model was prepared by injecting CAL27 or CAL27R (2 × 10^6^) cells into the right side of 6-week-old thymic nude mice (*n* = 5). The cell-injected nude mice were randomly grouped and different treatments were performed when the tumor volume reached 100 mm^3^. Mice (*n* = 5) were exposed to local IR by X-rays irradiated by X-RAD 320 (Precision X-Ray, Co., Ltd.) at a total dose of 10 Gy (2 Gy, 5 times). For the Akt inhibitor MK2206, nude mice were randomized into 4 groups (*n* = 5): 1, vehicle control (0.5% dimethyl sulfoxide, 100 µL/every 2 days, intraperitoneal injection); 2, local IR (2 Gy, 5 times); 3, MK2206 (20 mg/kg/every 2 days); and 4, local IR (2 Gy, 5 times) + MK2206 (20 mg/kg/every 2 days). Tumor volume was measured according to the formula (length×width^2^/2). Finally, the nude mice were euthanized with CO_2_ and tumor mass was taken to record the weight. All tumor mass and mouse organs, including spleen, kidney, lung, heart and liver tissues, were fixed in 4% formaldehyde and subjected to immunohistochemistry analysis.

### Statistical analysis

Statistical analyses were performed using GraphPad Prism software. Each experiment was guaranteed to have at least three independent measurements. The student’s t-test and one-way ANOVA statistical method were used between different groups. *p* < 0.05 indicates statistical significance.

## Results

### Correlation of Skp2 with malignant phenotype in OSCC

**Fig. 1:**
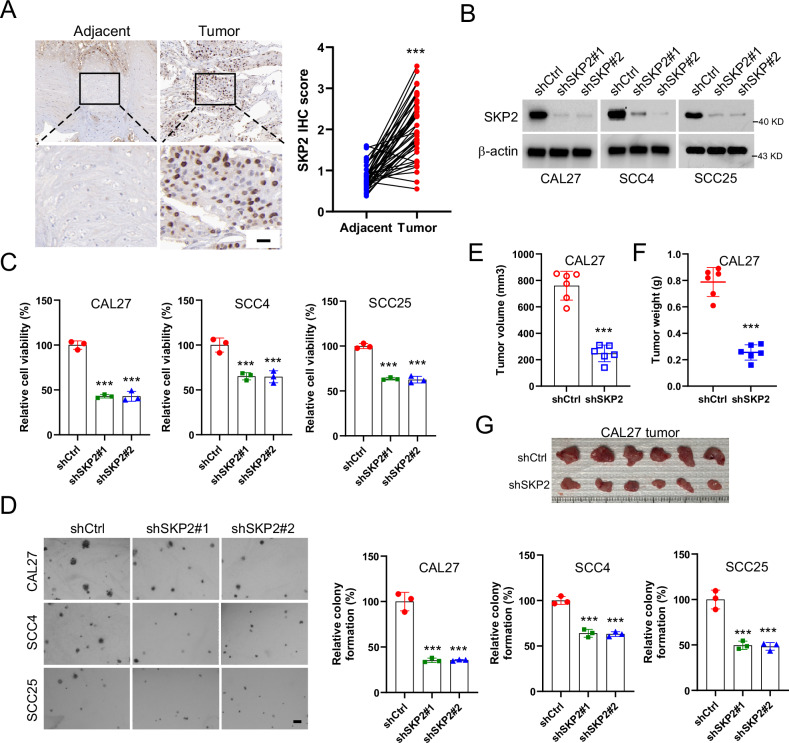
Skp2 is essential for maintaining the tumorigenicity of human oral squamous carcinoma cells. **A** IHC images of Skp2 expression in 40 cases of OSCC tumor and paracancerous specimens (left) and qualification (right). ***p < 0.001. Scale bar, 20 μm. **B** Immunoblotting (IB) examines Skp2 protein levels in Skp2 knockdown oral squamous carcinoma (OSCC) cells. **C** MTS assay detects cell viability. ***p < 0.001. **D** Soft agar colony formation assay was performed to detect colony formation after Skp2 knockdown. ***p < 0.001. Scale bar, 200 μm. **E**–**G** Xenograft tumor model was performed to analyze the effect of Skp2 knockdown on tumor volume (**E**), tumor weight (**F**), and tumor mass (**G**) of CAL27 tumors (*n* = 6). ***p < 0.001.

To examine the protein level of Skp2 in human oral squamous cell carcinoma, the immunohistochemical experiment was performed. Our results showed that the expression level of Skp2 was significantly upregulated in OSCC tissues (Fig. 1A). We constructed Skp2 knockdown stable cell lines in CAL27, SCC4, and SCC25 cells (Fig. 1B). We found that reduction of Skp2 expression suppressed the cell viability and colony formation in soft agar of three OSCC cells (Fig. 1C, D). We also tested the effect of Skp2 on tumorigenesis in vivo. Our results showed that the tumor volume and weight (Fig. 1E–G) significantly reduced in Skp2 knockdown xenografts. These results indicate that Skp2 is required to maintain the malignancy phenotype of OSCC cells.

### Skp2 depletion promotes apoptosis of OSCC cells under the effect of IR

**Fig. 2:**
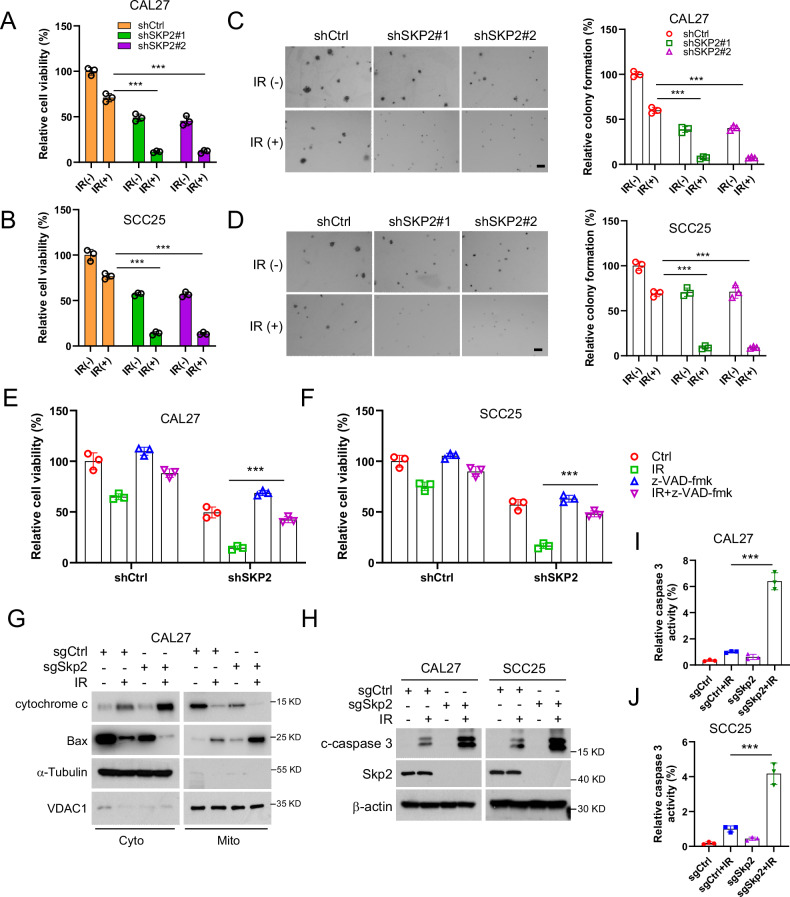
Skp2 knockdown enhances IR-induced OSCC-intrinsic apoptosis. **A**, **B** Skp2 knockdown CAL27 cells (**A**) and SCC25 cells (**B**) were assayed for cell viability by MTS after IR (2 Gy) treatment. ***p < 0.001. **C**, **D** Skp2 knockdown CAL27 cells (**C**) and SCC25 cells (**D**) were assayed for colony-forming ability by soft agar colony formation assay after IR (2 Gy) treated/untreated. ***p < 0.001. Scale bar, 200 μm. **E**, **F** Cell viability of shSkp2 CAL27 cells (**E**) /shSkp2 SCC25 cells (**F**) was assayed by MTS using apoptosis inhibitor (z-VAD-fmk) treatment. ***p < 0.001. **G**, **H** Skp2-knockdown CAL27 cells treated with/without IR (2 Gy) after 72 h, subcellular fractions (**G**) and whole cell extracts (**H**) were isolated for IB analysis. **I**, **J** Caspase 3 activity was examined by caspase 3 assay kit in CAL27 cells (**I**) and SCC25 cells (**J**). ***p < 0.001.

We next determined whether Skp2 knockdown affects the sensitivity of human OSCC cells to IR treatment. MTS experiments revealed that Skp2 knockdown reduced the cell viability of CAL27 and SCC25 cells, further enhancing the anti-tumor effect of IR treatment (Fig. 2A, B). Soft agar assay demonstrated that the colony formation of OSCC cells was further inhibited in the presence of IR (Fig. 2C, D). To clarify whether IR-suppressed OSCC cell viability is related to the activation of the apoptotic pathway, we pretreated OSCC cells with the pan-apoptosis inhibitor, z-VAD-fmk. As shown in Fig. 2E, F, the cell viability was partially restored by the apoptosis inhibitor z-VAD-fmk on IR-treated CAL27 and SCC25 cells, suggesting that IR treatment activated the apoptotic signaling. Moreover, the subcellular assay showed that the cytochrome C was released from mitochondria into the cytoplasm after IR treatment, while the protein expression level of Bax increased in mitochondria and decreased in the cytoplasm (Fig. 2G), indicating that IR promotes the intrinsic apoptosis. In addition, IB data showed a significant increase in both protein expression and relative activity of cleaved-caspase 3 after IR treatment (Fig. 2H–J). These results suggest that IR inhibited cell viability and promoted apoptosis in Skp2 knockdown OSCC cells.

### Downregulation of Survivin promotes apoptosis in Skp2-deficient OSCC cells

**Fig. 3:**
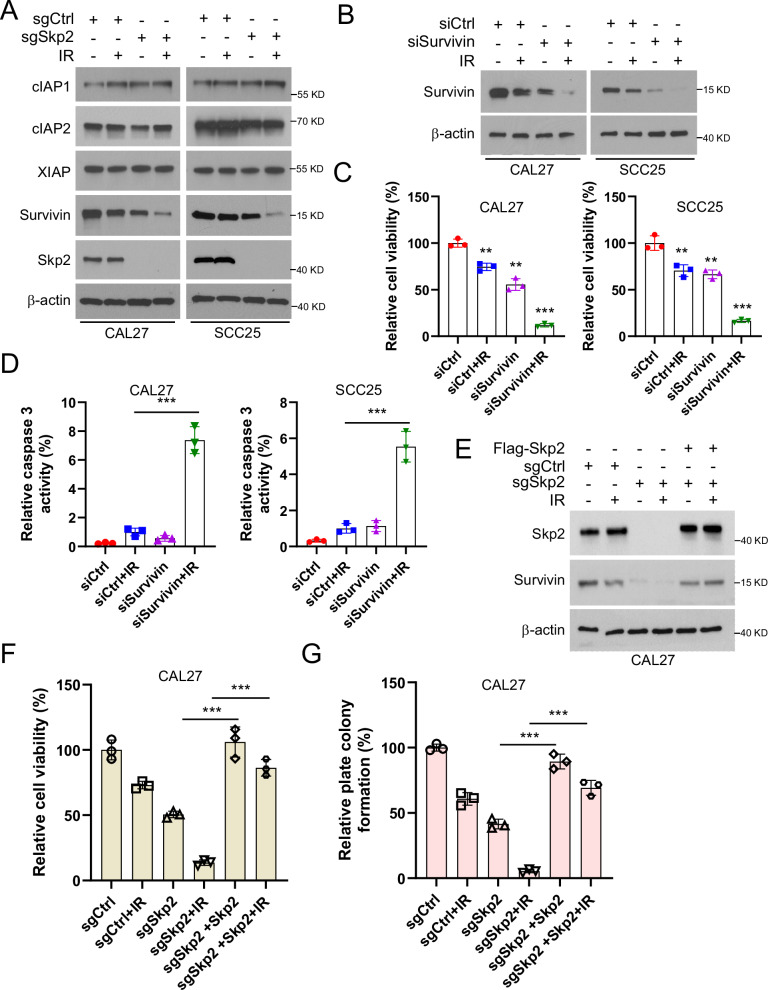
Skp2 deletion and IR treatment reduce Survivin protein levels. **A** Skp2 deficient OSCC cells were treated with/without IR and cultured for 24 h followed by IB assay. **B**–**D** CAL27 and SCC25 cells were transiently transfected with siRNA targeting Survivin for 24 h after transfection, followed by IR treatment and cultured for 24 h. **B** IB assay to detect the protein expression of Survivin. **C** MTS assay to detect cell viability. ***p < 0.001. **D** Caspase 3 activity was detected in CAL27 (left) and SCC25 (right) cells using caspase 3 assay kit. ***p < 0.001. **E**–**G** Flag-Skp2 was transfected in Skp2 knockdown CAL27 cells for 48 h, then treated with IR and cultured for 24 h. Protein expression levels of Skp2 and Survivin were detected by IB (**E**), cell viability was detected by MTS assay (**F**) and colony formation ability of OSCC cells was detected by colony formation assay (**G**). ***p < 0.001.

The inhibitor of apoptosis (IAP) family play crucial roles in anti-apoptosis [30]. We examined the protein levels of IAP family members in Skp2 knockout CAL27 and SCC25 cells. Our results showed that the IR treatment downregulates Survivin protein expression, with a more pronounced effect observed in Skp2 knockout cells (Fig. 3A). However, the other members of IAP family, including cIAP1, cIAP2, and XIAP, were unchanged. Next, we constructed Survivin gene silencing cell lines using siRNA (Fig. 3B). It was found that the cell viability was attenuated upon exposure to IR (Fig. 3C). Further studies revealed that caspase 3 activity was subsequently elevated after IR treatment in Survivin siRNA transfected cells (Fig. 3D). To determine the role of Skp2 in the maintenance of Survivin expression in oral squamous carcinoma cells, we transfected Flag-Skp2 into Skp2 knockout oral squamous carcinoma cells and found that Survivin protein level (Fig. 3E), cell viability (Fig. 3F), and the ability to form colonies on plates (Fig. 3G) could be restored after overexpression of Skp2 even with IR treatment. These results suggest that Skp2 is required for maintaining Survivin expression in oral squamous carcinoma cells.

### Skp2 deficiency promotes Survivin ubiquitination and degradation in a Thr34 phosphorylation-dependent manner

**Fig. 4:**
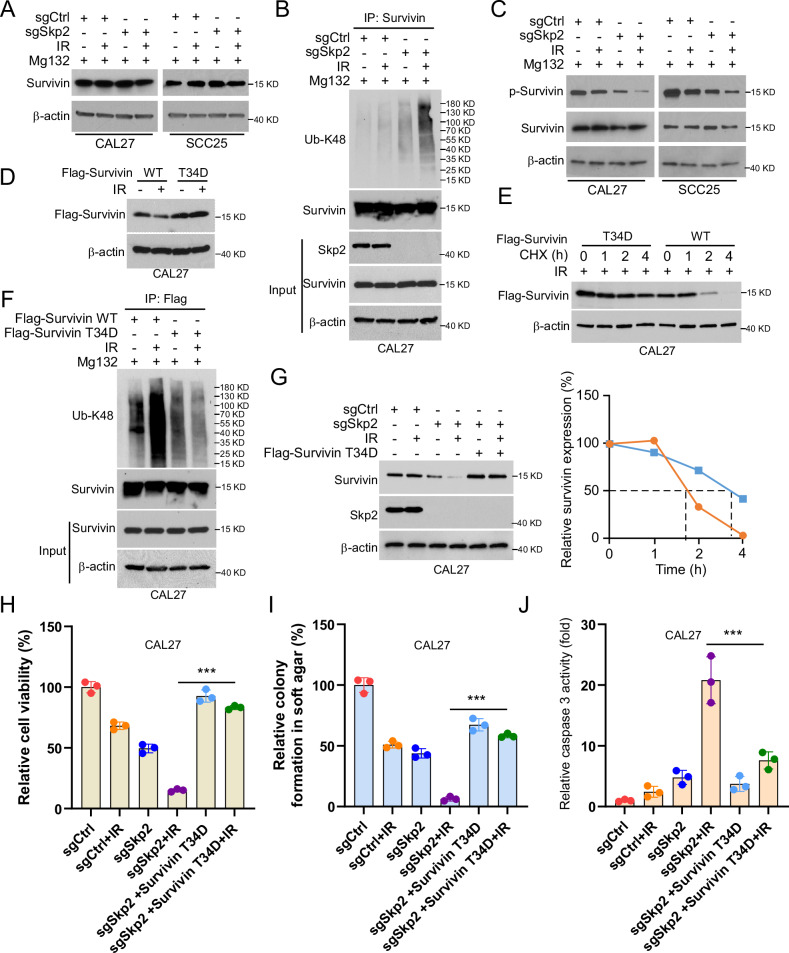
IR treatment promotes ubiquitination and degradation of Survivin in Skp2-deficient OSCC cells. **A** Skp2-deficient OSCC cells were treated with IR for 24 h and given 20 μM MG132 for 6 h, followed by IB assay. **B**, **C** (**B**) Immunoprecipitation (IP) was performed to determine the changes in the ubiquitination level of Survivin after co-treatment of Skp2-deficient OSCC cells with IR and MG132. **C** Immunoblotting experiments were performed to detect Survivin phosphorylation and total protein expression levels. **D** The protein expression level of Survivin was analyzed by IB assay after transfection of Flag-Survivin-WT/T34D into CAL27 cells for 48 h and IR treatment for 24 h. **E** The corresponding plasmids were transfected into OSCC cells for 48 h followed by IR treatment with CHX (20 μg/ml) for different incubation times. IB assay was performed on WCE to analyze the half-life of OSCC cells. Below, qualification for the above immunoblotting bands. **F** IP assay to determine the ubiquitination of Flag-Survivin-WT/T34D with IR treatment. **G** Skp2-deficient OSCC cells were transfected with Flag-Survivin-T34D for 48 h, treated with IR, and cultured for 24 h. WCE was subjected to analyze the protein levels of Survivin and Skp2 by IB. **H**–**J** MTS (**H**) assay, soft agar colony formation assay (**I**), and caspase 3 activity (**J**) were examined in Flag-Survivin-T34D transfected cells with IR treatment. ***p < 0.001.

To investigate the mechanism behind the downregulation of Survivin, we exposed Skp2-deficient CAL27 and SCC25 cells to a proteasome inhibitor (MG132) and observed that the presence of MG132 led to the stabilization of Survivin (Fig. 4A). The endogenous ubiquitination assay and IB results showed that Survivin K48-linked polyubiquitination (for proteosome degradation) was promoted in IR-treated Skp2-null CAL27 cells (Fig. 4B). The phosphorylation of Survivin on Thr34, a residue which required for Survivin stabilization [26], was decreased and weaken further in Skp2-null cells in the presence of IR (Fig. 4C). We constructed Survivin T34D mutants to mimic constitutively Survivin phosphorylation. Our results showed that the expression of Survivin (T34D) was not affected by IR treatment (Fig. 4D). In addition, the T34D mutation prolonged the half-life of Survivin (Fig. 4E) and reduced Survivin ubiquitination after IR treatment (Fig. 4F). Further result showed that the Survivin T34D mutation also restored Survivin protein expression levels (Fig. 4G), cell viability (Fig. 4H), colony formation (Fig. 4I), and compromised caspase 3 activity (Fig. 4J) with IR treatment.

### Skp2 agonizes FBXL7-mediated Survivin ubiquitination and degradation

**Fig. 5:**
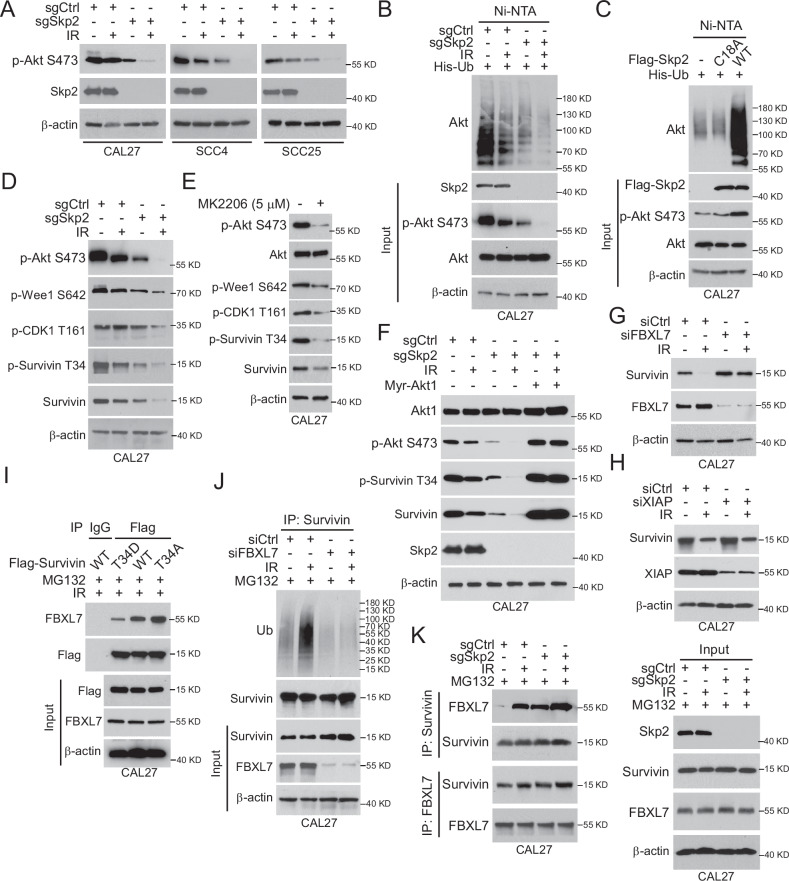
Skp2 regulation of the Akt/Wee1 signaling axis is required for the maintenance of Survivin stabilization. **A** Skp2-deficient OSCC cells were treated with/without IR and subsequently analyzed by IB. **B** Akt ubiquitination was detected by Ni-NTA Pull-down assay. **C** Flag-Skp2 WT or C18A mutants and His-Ub were transfected into OSCC cells for 48 h followed by Ni-NTA Pull-down experiment. **D** IB assay for the effect of IR on the Akt/Wee1 signaling axis in Skp2 deficient cells. **E** IB assay of the Akt inhibitor (MK2206 5 μM) on the Akt/Wee1 signaling axis in CAL27 cells. **F** IB analysis was performed in Skp2-deficient CAL27 cells transfected with Mry-Akt1 plasmid for 48 h, followed by IR treatment for 24 h. WCE was prepared for IB analysis. **G**, **H** CAL27 cells were transfected with siCtrl, siFBXL7 (**G**) or siXIAP (**H**) for 24 h and treated with/without IR, followed by IB analysis. **I** Flag-Survivin-WT or T34D was transfected into CAL27 cells for 48 h. IR treatment was performed and cells were cultured for 24 h, MG132 was added to the culture medium for 6 h. The WCE was subjected to IB analysis. **J** IP assay to detect the ubiquitination of Survivin in OSCC cells after siFBXL7 transfection and IR treatment. **K** Co-Immunoprecipitation (Co-IP) was performed to detect the interaction between FBXL7 and Survivin in Skp2-deficient CAL27 cells.

Survivin Thr34 phosphorylation is regulated by cdc2, a downstream kinase of Akt signaling [26]. To investigate whether Skp2 affects Akt signaling in OSCC after IR treatment, we examined the protein and expression of Akt phosphorylation on Ser473 in Skp2-null OSCC cells. The results showed a substantial reduction of Akt phosphorylation with irradiation (Fig. 5A). As Akt non-degradative ubiquitination by Skp2 is required for Akt activation in EGF signaling [14], we determined whether reduced Akt phosphorylation is related to Akt ubiquitination modification. The result showed that Akt ubiquitination level is attenuated in sgSkp2-CAL27 cells upon exposure to IR (Fig. 5B). Moreover, Skp2 wild type (WT), but not the catalytic inactive mutant C18A, promoted Akt polyubiquitination (Fig. 5C). Skp2 deletion decreased the phosphorylation of Wee1 (Ser642), CDK1 (Thr161), and Survivin (Thr34), as well as total Survivin protein levels in IR-treated CAL27 cells (Fig. 5D). In addition, the Akt inhibitor (MK2206) reduced total Survivin levels as well as the phosphorylation of Wee1 CDK1 Thr161, Wee1 Ser642 and Survivin Thr34 (Fig. 5E). Further elucidation of the mechanism of the Akt pathway on Survivin in OSCC, we revealed that constitutively activated Myr-Akt1 overexpression rescued the reduced phosphorylation and protein expression of Survivin (Thr34) after IR treatment (Fig. 5F). All of these results suggested that the Skp2-Akt signaling is required for Survivin stability in IR-treated OSCC cells. FBXL7 and XIAP, are two E3 ligases [31, 32] that degrade Survivin in human cells. Our data showed that only silence FBXL7 restored Survivin expression (Fig. 5G, H). The IB results showed the weakest interaction between Flag-Survivin T34D and FBXL7, suggesting that phosphorylation of Thr34 occurs to attenuate the interaction between Survivin and FBXL7 (Fig. 5I). The endogenous ubiquitination results showed that Survivin ubiquitination was reduced after FBXL7 silencing (Fig. 5J). In addition, the interaction present between FBXL7 and Survivin was further increased in Skp2 knockout cells with IR treatment (Fig. 5K). These data suggest that inhibition of Akt signaling leads to Survivin degradation in Skp2-null OSCC cells.

### Skp2 depletion promotes the radiosensitivity of OSCC cells in vivo

**Fig. 6:**
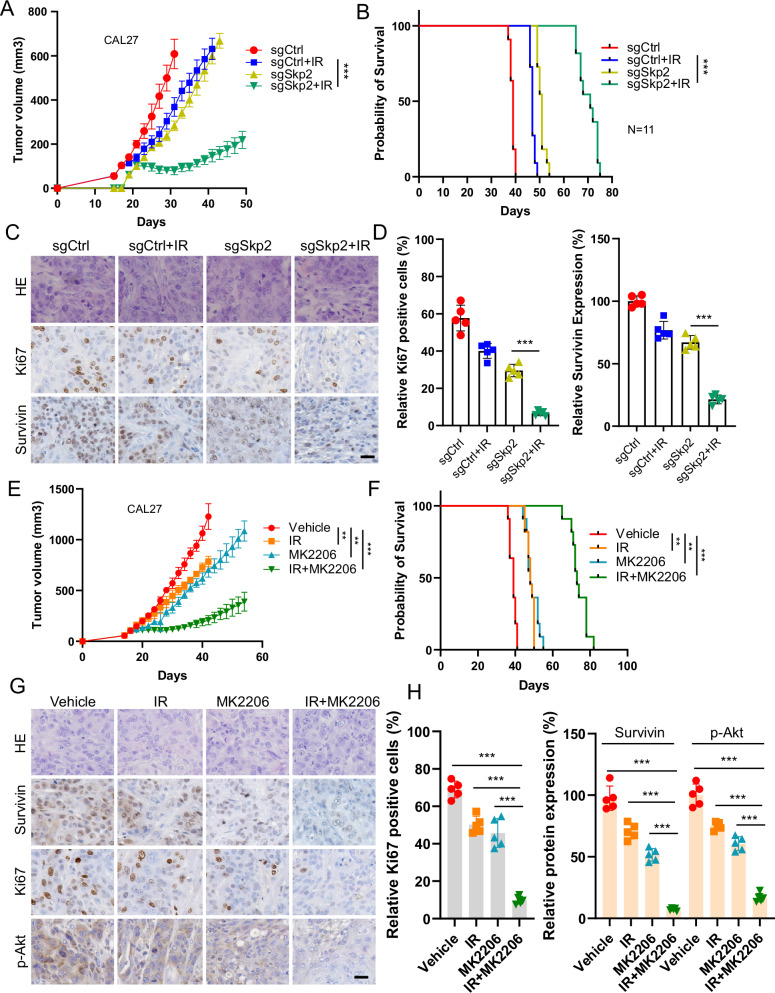
Skp2-Akt signaling confers radioresistance of OSCC Cells. **A**–**D** CAL27 cell-derived tumors were treated with IR. Tumor volume (**A**) and mice survival time (**B**) were recorded. Tumor tissues were collected and subjected to IHC staining (**C**, Scale bar, 20 μm). **D** Qualification of (**C**). ***p < 0.001. **E**–**H** CAL27 cell-derived tumors were treated with IR, MK2206, or combination. Tumor volume (**E**) and mice survival time (**F**) were recorded. Tumor tissues were collected and subjected to IHC staining (**G**, Scale bar, 20 μm). **H** Qualification of (**G**). **p < 0.01, ***p < 0.001.

We used the Skp2 knockout CAL27 stable cells to establish a xenograft tumor model. The results demonstrated that the Skp2 knockout xenograft tumors exhibited a slower tumor growth efficacy, which became smaller with IR treatment (Fig. 6A). Moreover, after radiation treatment, the survival time of mice with Skp2 knockout xenograft tumors was prolonged (Fig. 6B). The results of IHC staining suggested that the expression levels of Ki67 and Survivin were reduced with Skp2 knockout or radiotherapy. The effect of combined radiotherapy was more significant than the Skp2 proficient-, deficient- tumors, or only radiotherapy tumors (Fig. 6C, D). As shown in Figure E and F, IR or MK2206 monotherapy inhibited the growth of CAL27 tumors (Fig. 6E), prolonged the survival time of mice (Fig. 6F), and was further increased by combined radiotherapy. Immunohistochemical results showed that MK2206 treatment/IR treatment alone reduced the expression of Ki67, Survivin and Akt phosphorylation, which was further enhanced by combined treatment (Fig. 6G, H).

### Skp2 was positively correlated with Survivin in OSCC tissues

**Fig. 7:**
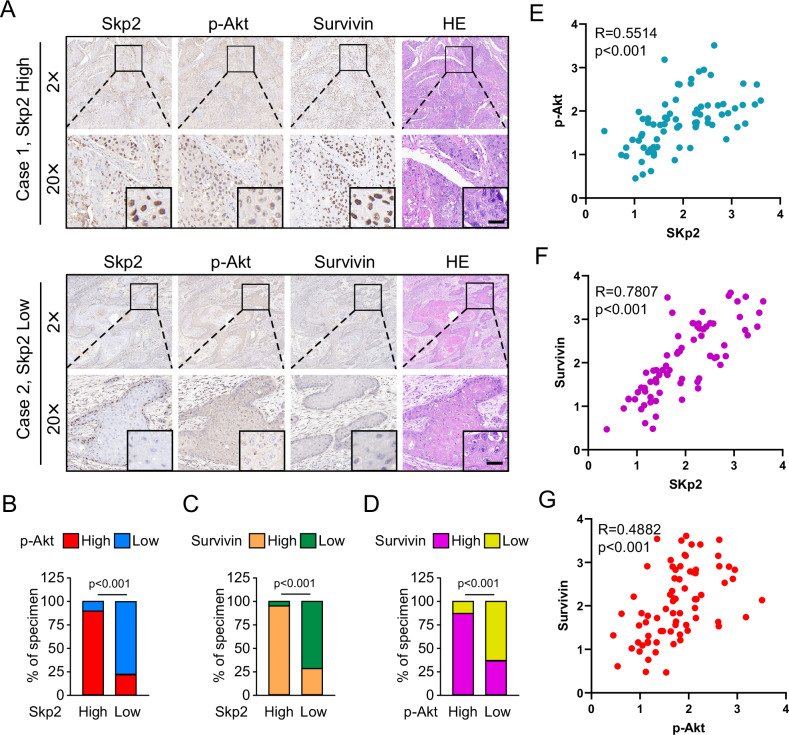
Skp2 is overexpressed and positively correlated with Survivin and p-Akt in OSCC tissues. **A** Representative IHC staining images of tumor tissues from OSCC patients with high levels of Skp2, p-Akt Survivin protein or low levels of Skp2, p-Akt Survivin protein. Scale bar, 20 μm. **B**, **C** Percentage of samples with high or low Skp2 expression compared with p-Akt (**B**) and Survivin (**C**) expression levels. **D** Percentage of samples with high or low p-Akt expression compared to the expression levels of Survivin. **E**–**G** Scatter plots showing the positive correlation between p-Akt and Skp2 (**E**), Survivin and Skp2 (**F**), and Survivin and p-Akt (**G**) expression in OSCC tumor tissues, respectively.

To further elucidate whether our study is clinically relevant. We assessed the expression of Skp2, p-Akt and Survivin in the tumor tissues of 74 patients by IHC analysis. The results were as follows, and representative IHC images of low and high expression levels of Skp2, p-Akt, and Survivin are illustrated in Fig. 7A. We found that out of 74 patients, 48 tumor tissues showed high levels of Skp2 staining, and out of these 48 cases, 44 also showed high levels of p-Akt staining, and 46 also showed high levels of Survivin staining (Fig. 7B, C). In addition, p-Akt was detected as highly expressed in the tumor tissue of 50 patients out of all the study subjects, and 44 of these 50 cases also had high Survivin staining (Fig. 7D). Unsurprisingly, we also observed statistically significant positive correlations between Skp2 and p-Akt, Survivin, and between p-Akt and Survivin (Fig. 7E–G). We further utilized GSE30784 and GSE31056 from GEO datasets associated with OSCC to analyze the correlation among Skp2, Akt, and Survivin in OSCC tissues. The results from both datasets indicated a positive correlation between Skp2 and both Akt and Survivin and a positive correlation between Akt and Survivin (Fig. S1). Altogether, these data suggested that all three were overexpressed and positively correlated with each other in oral squamous cell carcinoma tissues.

### Skp2 is overexpressed in radioresistant OSCC cells

**Fig. 8:**
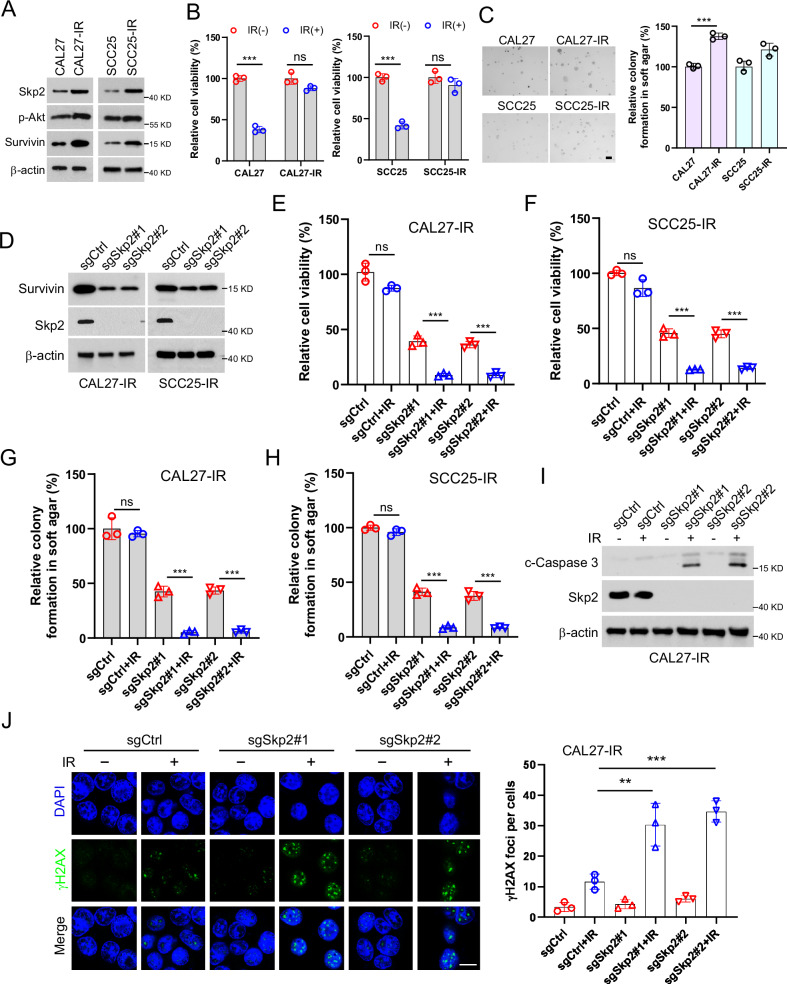
Skp2 is overexpressed in radioresistant OSCC cells. **A** WCE of CAL27/CAL27-IR and SCC25/SCC25-IR cells were subjected to IB analysis. **B** CAL27/CAL27-IR and SCC25/SCC25-IR cells were treated with/without IR and analyzed using the MTS assay. ns: no statistical significance. ***p < 0.001. **C** Soft agar colony formation assay was performed and statistically analyzed on CAL27/CAL27-IR and SCC25/SCC25-IR cells. *p < 0.05. ***p < 0.001. Scale bar, 200 μm. **D** IB was performed in Skp2-deficient CAL27-IR and SCC25-IR cells to detect Survivin protein expression levels. **E**, **F** MTS analysis of cell viability of CAL27-IR (**E**) and SCC25-IR cells (**F**) with IR treatment. **G**, **H** Colony formation of CAL27-IR (**G**) and SCC25-IR (**H**) cells with IR treatment. ***p < 0.001. **I**, **J** CAL27-IR cells with IR treatment after knockout of Skp2. Cleaved-caspase 3 protein were detected by IB analysis (**I**) and γ-H2AX was determined by immunofluorescence (**J**, left, Scale bar, 10 μm). Right, qualification. **p < 0.01, ***p < 0.001.

The aforementioned data suggest that Skp2 correlates with OSCC radiotherapy sensitivity. To further validate the mechanism of Skp2 in OSCC radiotherapy resistance, we examined the protein expression level of Skp2 in two pairs of radioresistant cell lines. The results showed that Skp2 was highly expressed in the resistant CAL27-IR and SCC25-IR cells compared with the parental sensitive cells, CAL27 and SCC25 (Fig. 8A). The MTS experiments illustrated that, after IR treatment, the radiotherapy-resistant cells did not show significant changes in viability (Fig. 8B). The results of soft agar colony formation assay showed that the radiotherapy-resistant cell line had stronger colony formation ability (Fig. 8C). We found that the protein expression of Survivin was reduced in Skp2-null CAL27-IR and SCC25-IR cells (Fig. 8D). In addition, both cell viability and colony formation ability were significantly reduced in Skp2 knockout resistant cells, and this effect was further enhanced by combined radiotherapy (Fig. 8E–H). IB data showed elevated cleaved-caspase 3 protein levels in Skp2-deficient SCC25-IR cells after exposure to IR (Fig. 8I). Immunofluorescence experiments revealed that the enhanced DNA damage signaling, the staining of γ-H2AX, was observed in Skp2-null cells (Fig. 8J), indicating that Skp2 deficiency resensitizes IR treatment.

### Inhibition of Skp2 overcomes radioresistance of OSCC cells

**Fig. 9:**
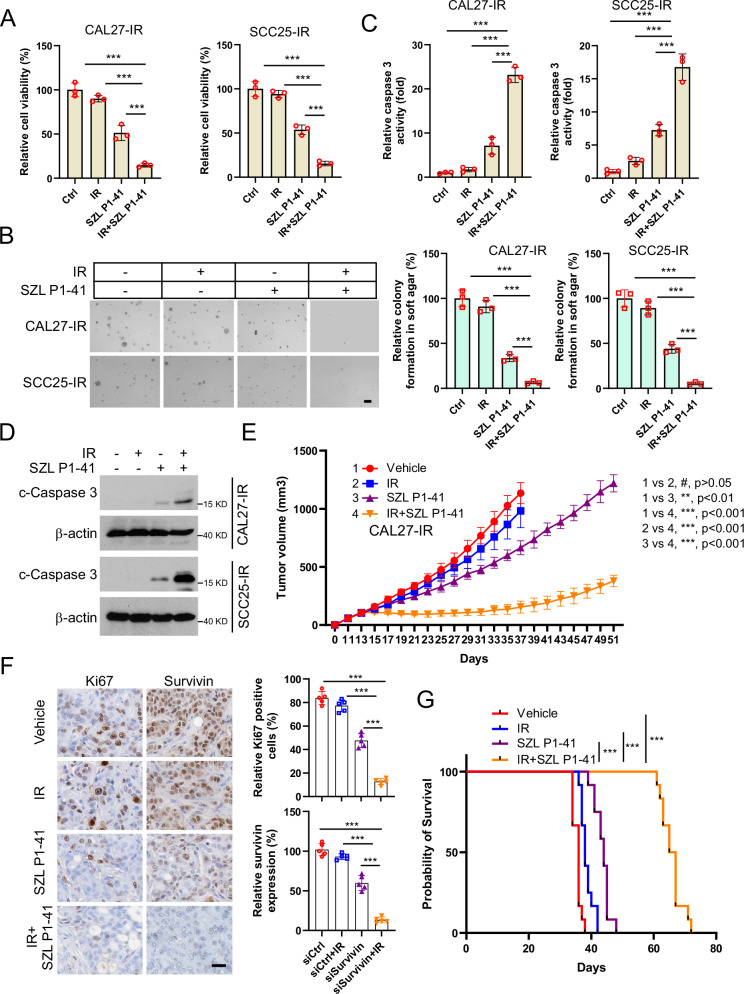
Skp2 inhibitor use partially overcomes radiotherapy resistance in OSCC cells. **A**–**D** CAL27-IR and SCC25-IR cells were subjected to IR, SZL PI-41, or co-treatment and were assayed for cell viability using MTS (**A**), colony formation using soft agar colony formation assay (**B**), Scale bar, 200 μm), caspase 3 activity using caspase 3 assay kit (**C**), and IB for cleaved-caspase 3 protein expression level (**D**). ***p < 0.001. **E**–**G** CAL27-IR cell-derived tumors were treated with IR, SZL PI-41 or combination therapy. Tumor volume (**E**) and mouse survival time (**G**) were recorded. Tumor tissues were collected and stained with IHC (**F**, Scale bar, 20 μm). **P < 0.01, ***P < 0.001.

Previous reports have identified SZL P1-41 as a Skp2-specific inhibitor [33, 34]. We treated radiotherapy-resistant cell lines with SZL P1-41 and found that SZL P1-41 decreased the cell viability (Fig. 9A) and colony-forming ability (Fig. 9B) of the radiotherapy-resistant cells. The activity of caspase 3 (Fig. 9C) and the protein level of cleaved-caspase 3 in radioresistant cells were increased (Fig. 9D), and all of these above effects were further enhanced after combined radiotherapy treatment. In the xenograft tumor model, it was observed that SZL P1-41, rather than IR, decreased tumor growth and volume in vivo, with the combined treatment enhancing this anti-tumor effectiveness (Fig. 9E). IHC results revealed that the expression of Ki67 and Survivin were significantly reduced by SZL P1-41 and radiotherapy combination (Fig. 9F). In addition, as shown in Fig. 9G, combined treatment with SZL P1-41 and IR notability increased the survival of mice. These results suggest that SZL P1-41 treatment overcomes radioresistance in OSCC.

## Discussion

Oral squamous cell carcinoma (OSCC) is highly aggressive and heterogeneous [1]. Although surgical resection supplemented by radiotherapy is improving as an important treatment modality for OSCC treatment, the prognosis of patients with advanced OSCC is still poor, and the existence of resistance to radiotherapy remains an urgent challenge [2, 35]. Currently, studies have reported that P63, methyltransferase-like 3 (METTL3), and integrin β1 (ITGB1) can be used as biomarkers of radiation resistance in OSCC [36–38], and are expected to be promising therapeutic targets for OSCC. In addition, neuronal precursor cell-expressed developmental down-regulated protein 8 (NEDD8) can restore OSCC sensitivity to radiation therapy by triggering autophagy formation [39]. AIM2 can promote OSCC radioresistance and metastasis [40]. Blocking IL-6 signaling can restore OSCC sensitivity to radiation therapy [41]. However, the ubiquitination-related molecules associated with radiotherapy resistance in oral squamous carcinoma remain unclear. Therefore, further exploration regarding biomarkers and potential mechanisms of radiotherapy resistance is needed. Our study showed that Skp2 was highly expressed in OSCC radiotherapy-resistant cell lines (Fig. 8A). Further studies revealed that Skp2 deficiency restored the sensitivity of OSCC cells to radiotherapy, and our study suggests that Skp2 is a potential therapeutic target predictive of overcoming and treating OSCC.

S-phase kinase-associated protein 2 (Skp2) is a critical E3 ligase that recognizes substrates and thus promotes their ubiquitinated degradation to regulate various signaling pathways such as Wnt/β-catenin, p21/p27 and p53 pathways, thereby involving in many pathophysiological processes such as cell cycle, apoptosis, cell proliferation, and therapeutic resistance in tumor [42–46]. Skp2 is highly expressed in various malignancies and correlated with tumorigenesis and therapeutic resistance of OSCC [47], and thus, Skp2 is considered a potential therapeutic target for a wide range of tumors. Previous studies have shown that Skp2 has been shown to target phosphorylated p27 for proteasomal degradation, with regulatory effects on various cell biological functions [48]. For example, Skp2 promotes the ubiquitination and degradation of p27, inducing tumor cells to enter the S phase or transition from G1 to S phase, thereby increasing the proliferative capacity of cancer cells [44]. Moreover, it has been reported that Skp2-mediated ubiquitination and degradation of p27 require Cks1, and overexpression of Cks1 promotes radiotherapy resistance in esophageal squamous cell carcinoma [49]. Furthermore, down-regulating Skp2 promotes p21/p27 accumulation, resulting in cell cycle arrest and sensitive enhancement to treatment by inhibiting DNA damage repair [44, 48, 50]. In addition, previous studies also demonstrated that Skp2 promotes ubiquitination degradation of MLKL in non-small cell lung cancer, thereby overcoming cisplatin resistance [51]. Skp2 deficiency promotes Mcl-1 ubiquitination and degradation through the FBW7 pathway and improves the sensitivity of colorectal cancer (CRC) to IR [9]. In our study, we investigated the mechanism of Skp2 regulation of radiotherapy resistance in OSCC cells and found that Skp2 regulated cell viability and colony formation of OSCC cells (Fig. 1). In addition, our mechanistic studies revealed that Skp2 deficiency decreases the level of Akt ubiquitination, which in turn blocks the Akt/Wee1/CDK1/Survivin signaling pathway as a means of restoring the sensitivity of OSCC cells to radiation therapy.

Survivin regulates essential intracellular physiological functions and can inhibit tumor cell apoptosis, promote tumor cell proliferation, and tumor cell autophagy and tumor mesenchymal angiogenesis [52–55]. Therefore, Survivin has become an ideal tumor gene diagnosis and treatment target. Survivin is highly expressed in most tumor tissues, and its abnormally high expression is closely related to chemotherapy resistance, tumor recurrence and prognosis [56]. For example, targeting Survivin expression has been reported to modulate drug resistance in Acute myeloid leukemia (AML) and drug-resistant acute lymphoblastic leukemia (ALL) [57, 58]. In addition, Survivin knockdown induces apoptosis in cervical cancer cells and thus promotes the sensitivity of cervical cancer cells to radiation therapy [59]. High Survivin expression promotes HNSCC tumor growth and confers radiotherapy resistance [60]. In our study, both Survivin and Skp2 were highly expressed in oral squamous carcinoma tissues, and their expressions were positively correlated (Fig. 7). Our results indicated that Skp2 maintained Survivin expression in OSCC cells and could inhibit its ubiquitination degradation (Fig. 4). Thus, promoting Survivin degradation could be a promising strategy for OSCC cell radiosensitization, and targeting Survivin is essential for overcoming OSCC cell radiosensitization resistance.

## Conclusions

In conclusion, our results suggest that Skp2 has a crucial regulatory role in the mechanism of radiotherapy resistance in OSCC cells by activating the Akt/Wee1 signaling axis and thus stabilizing Survivin. Therefore, targeting Skp2-Akt-Survivin in combination with conventional radiotherapy will improve the therapeutic efficacy in oral squamous carcinoma.

## Supplementary information

supplemental figures and figure legends

supplementary file-full gels

## Data Availability

All data generated or analysed during this study are included in this published article (and its supplementary information files).
