# Supplementary material for: E3 ligase Skp2-mediated stabilization of survivin contributes to radioresistance
Source: Cell Death Discov. 2025 Apr 7;11:151. doi: 10.1038/s41420-025-02463-3 (PMC11977269; doi:10.1038/s41420-025-02463-3)
Supplement: Supplementary file 1 — supplemental figures and figure legends [file 41420_2025_2463_MOESM1_ESM.pdf]

Figure S1

A GSE30784

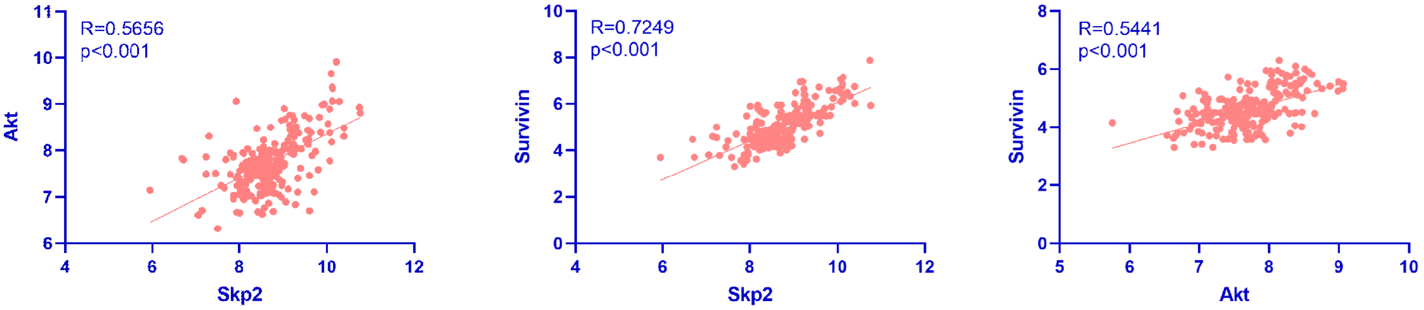

B GSE31056

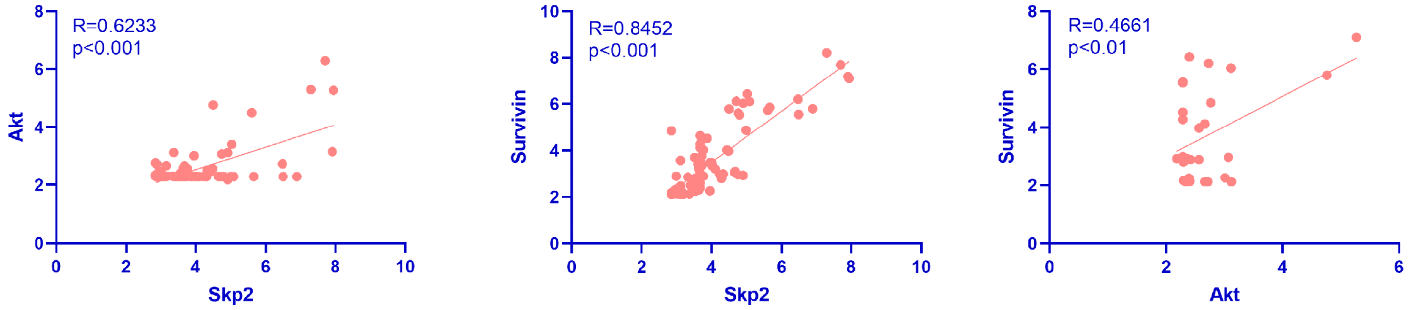

Supplementary Figure 1: (A-B) GSE30784 and GSE31056 datasets from GEO were used to analyze the correlation among Skp2, Akt, and survivin in OSCC tissues.
