# Supplementary material for: E3 ligase Skp2-mediated stabilization of survivin contributes to radioresistance
Source: Cell Death Discov. 2025 Apr 7;11:151. doi: 10.1038/s41420-025-02463-3 (PMC11977269; doi:10.1038/s41420-025-02463-3)

Full gel for Figure 1

B

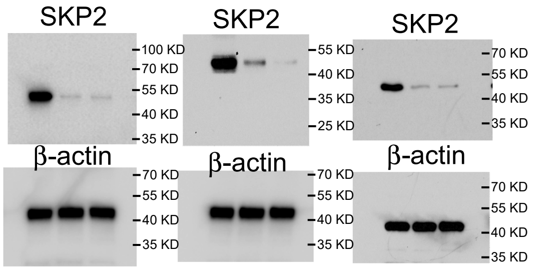

Full gel for Figure 2

G

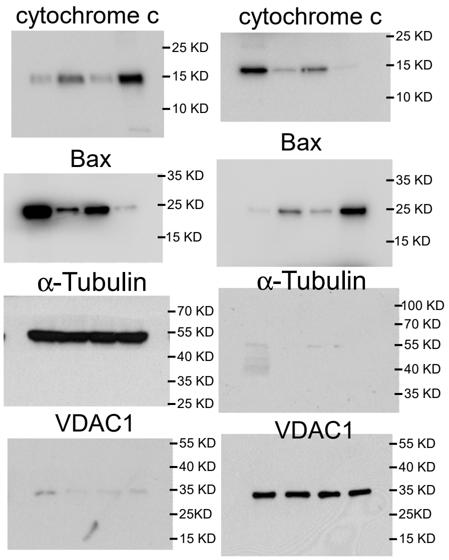

H

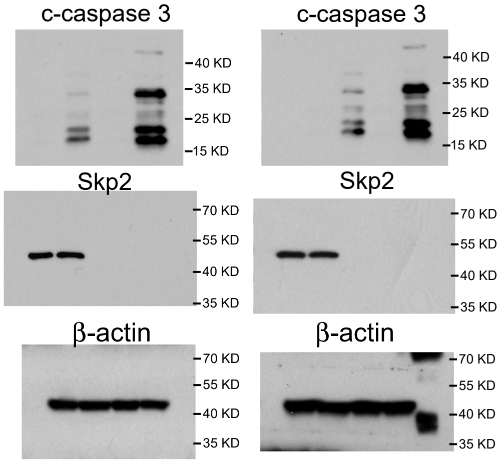

Full gel for Figure 3

A

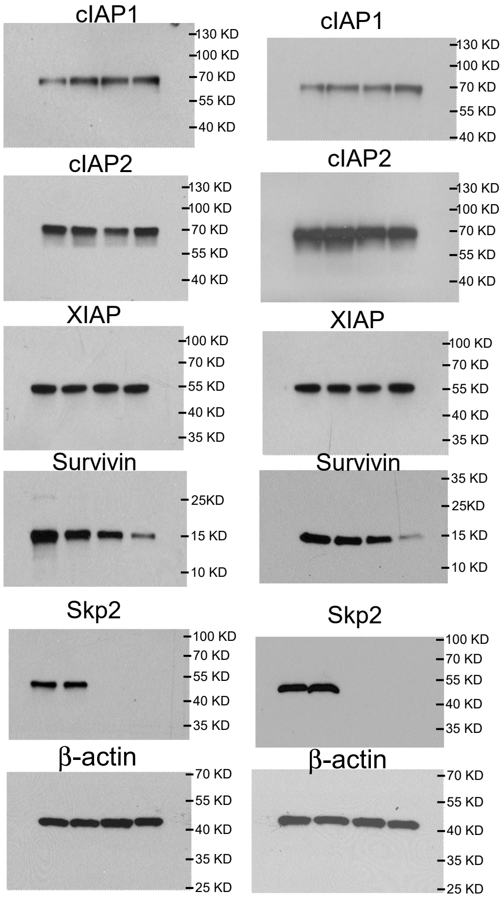

B

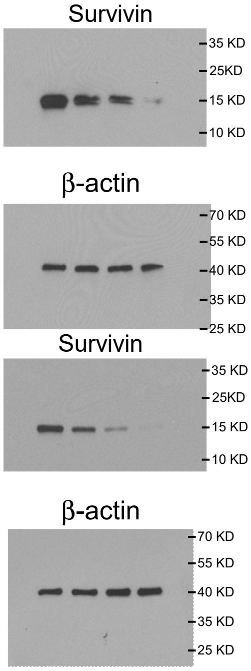

E

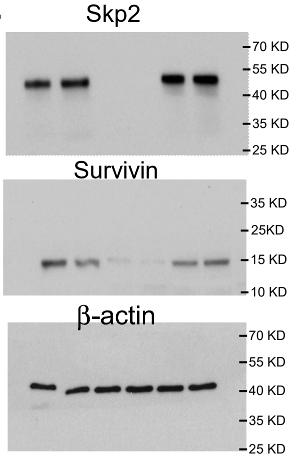

Full gel for Figure 4

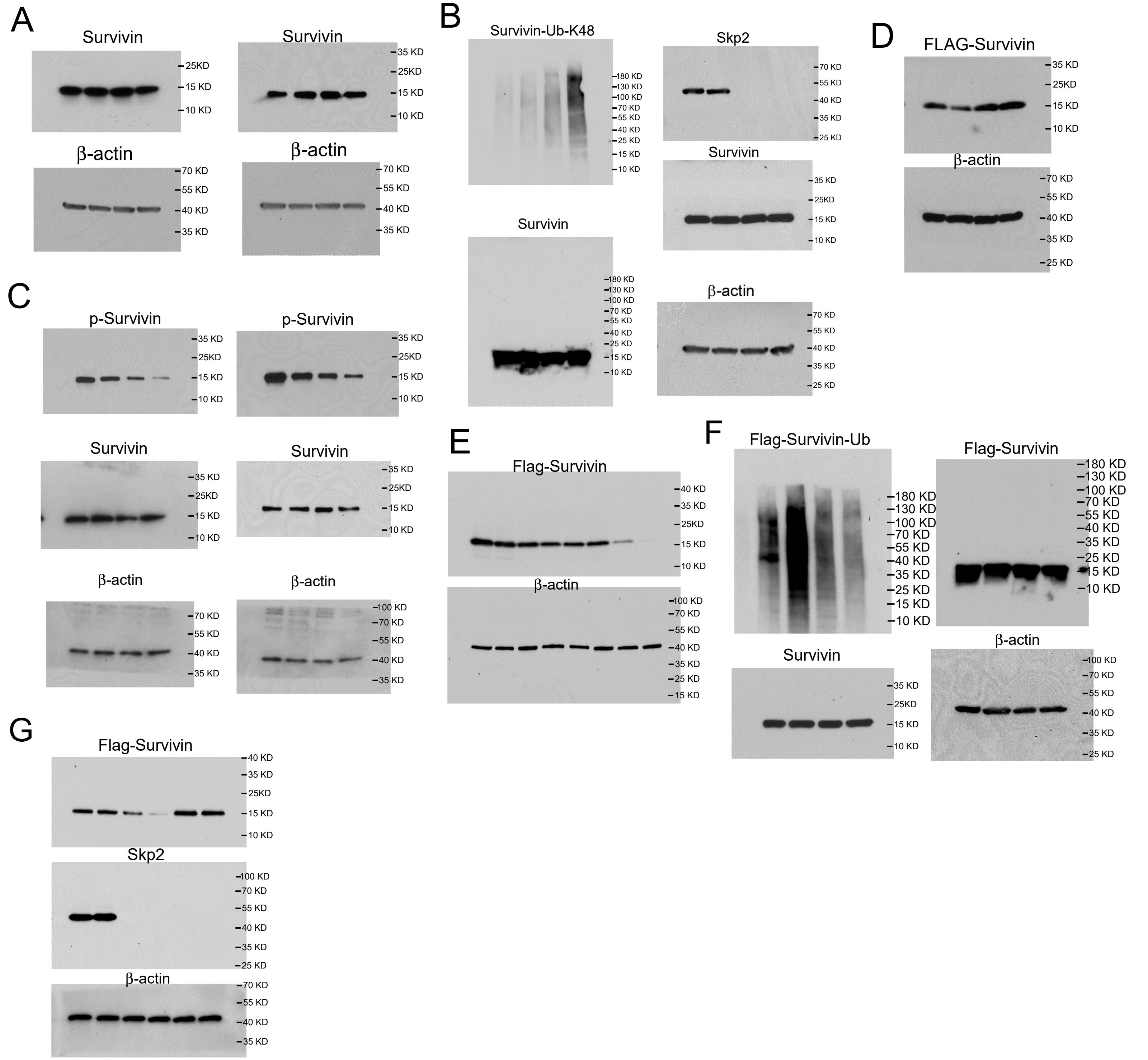

Full gel for Figure 5

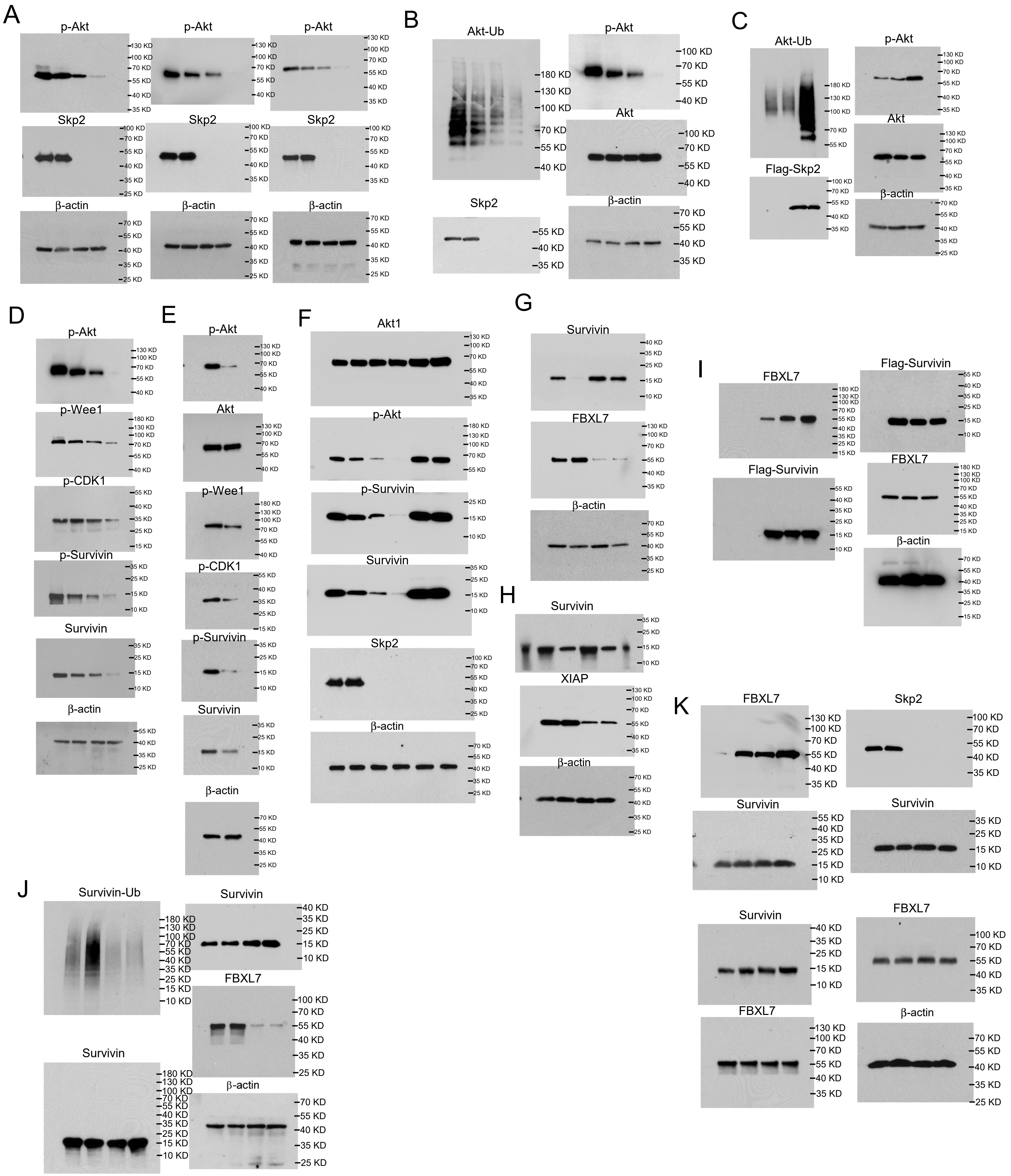

Full gel for Figure 8

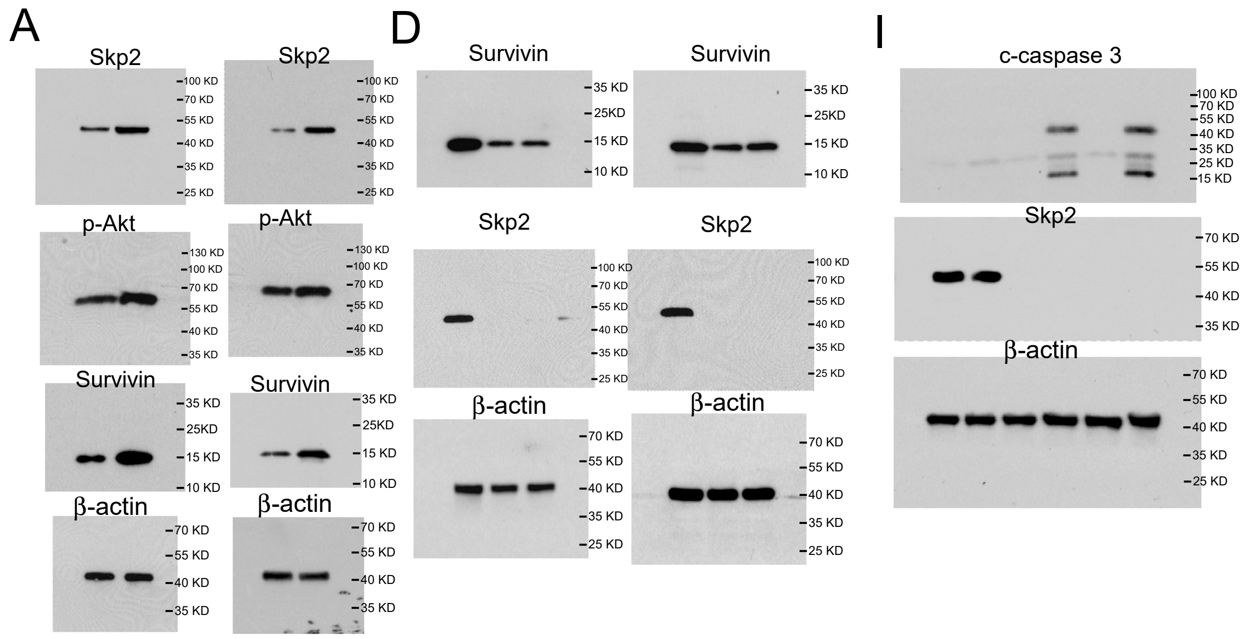

Full gel for Figure 9

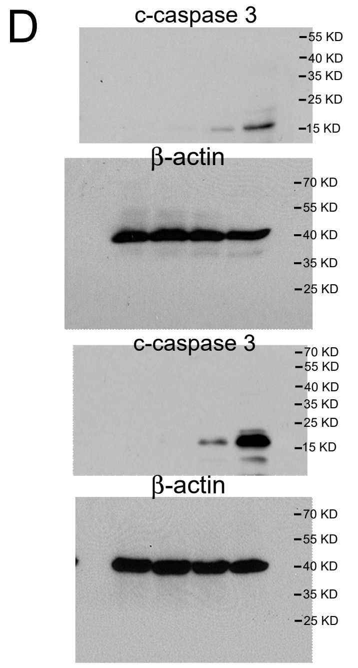

Supplement: Supplementary file 2 — supplementary file-full gels [file 41420_2025_2463_MOESM2_ESM.pdf]
